# ﻿New species and new records of *Scolytoplatypus* Schaufuss (Curculionidae, Scolytinae) from China, and resurrection of *Scolytoplatypussinensis* (Tsai & Huang, 1965) as a distinct species

**DOI:** 10.3897/zookeys.1082.77637

**Published:** 2022-01-18

**Authors:** Song Liao1, Shengchang Lai*, Roger A. Beaver, Heiko Gebhardt, Jianguo Wang

**Affiliations:** 1 Laboratory of Invasion Biology, Jiangxi Agricultural University, Nanchang, Jiangxi 340045, China Jiangxi Agricultural University Nanchang China; 2 College of Forestry, Nanjing Forestry University, Nanjing, Jiangsu 210037, China Nanjing Forestry University Nanjing China; 3 161/2 Mu 5, Soi Wat Pranon, T. Donkaew, A. Maerim, Chiangmai 50180, Thailand Unaffiliated Chiangmai Thailand; 4 Maienfeldstraße 23/1, D-72074 Tübingen, Germany Unaffiliated Tübingen Germany

**Keywords:** Ambrosia beetles, Fujian, Jiangxi, molecular phylogeny, Scolytoplatypodini, taxonomy

## Abstract

This study describes two new species, *Scolytoplatypuswugongshanensis* Liao, Lai & Beaver, **sp. nov.** and *S.skyliuae* Liao, Lai & Beaver, **sp. nov.**, reinstates *S.sinensis* (Tsai & Huang, 1965) from synonymy with *S.mikado* (Blandford, 1893), and records five species for the first time from China, *S.brahma* Blandford, 1898, *S.curviciliosus* Gebhardt, 2006, *S.minimus* Hagedorn, 1904, *S.ruficauda* Eggers, 1939, *S.samsinghensis* Maiti & Saha, 2009, and three from mainland China, *S.blandfordi* Gebhardt, 2006, *S.calvus* Beaver & Liu, 2007, *S.pubescens* Hagedorn, 1904. A key to the males of *Scolytoplatypus* species in China is given. Genetic data from four genes indicate a rather isolated position for both new species, although their genetic relationship to each other was close.

## ﻿Introduction

The genus *Scolytoplatypus* was erected for a single species, *S.permirus* Schaufuss, from Madagascar (Schaufuss 1891). To date, there are approximately 50 species reported in the world, most of which are distributed in the Afrotropical and Oriental regions, and a few in the temperate areas of Japan to India (Wood and Bright 1992; Bright and Skidmore 1997, 2002; Beaver and Liu 2007; Maiti and Saha 2009; Jordal 2013, 2018). Approximately 16 species are known from the Afrotropical region, including 12 or 13 species on the African continent and three species in Madagascar (Jordal 2018). Beaver and Gebhardt (2006) reviewed the genus in the Oriental region and recognised 28 species, six of which are known from China. Beaver and Liu (2007) described a new species *S.calvus* Beaver & Liu, from Taiwan. Knížek (2008) described a new species, *S.zahradniki* Knížek, from Shaanxi, China, and *S.darjeelingi* Stebbing was listed for Hunan, Sichuan, and Yunnan in Knížek (2011). The following nine species are currently recognised to occur in China: *S.blandfordi* Gebhardt, 2006; *S.calvus* Beaver & Liu, 2007; *S.darjeelingi* Stebbing, 1914; *S.mikado* (Blandford, 1893); *S.pubescens* Hagedorn, 1904; *S.raja* Blandford, 1893; *S.tycon* Blandford, 1893; *S.superciliosus* Tsai & Huang, 1965 and *S.zahradniki* Knížek, 2008. *S.blandfordi*, *S.calvus* and *S.pubescens* were found only in Taiwan, China.

All known species of *Scolytoplatypus* are ambrosia beetles (Beaver and Gebhardt 2006) which cultivate fungi in a gallery system as the only food source for larvae and adults. The females of most species have a unique mycangial structure for carrying fungal spores, located on the pronotum. However, this is absent in some species (Beaver and Gebhardt 2006), and the mechanism of ambrosial fungal transport is not known in these species (Mayers et al. 2020). In addition to the structural differences of the pronotum, the two sexes also show differences in the morphology of the frons, prosternum, protibia, and elytral declivity. The beetles are monogamous. The gallery system is started by the female. It consists of an entrance gallery leading to one or more circumferential branches in one transverse plane. The male joins the female soon after the beginning of maternal gallery construction, and mating occurs at the gallery entrance. The male remains in the entrance hole to prevent the entry of predators and helps with removal of the faecal material (Browne 1961). The eggs are laid in individual niches above and below the gallery. The larvae develop in individual barrel-shaped cells, feeding on the ambrosial fungus growing on the walls, and enlarging the cell as they grow. The fully grown larva pupates in the cell, and the new adult emerges into the gallery and leaves by the original entrance hole (Beeson 1961; Browne 1961; Beaver et al. 2014)

In recent years, we have made extensive collections of beetles in China. In this paper, we describe two new species of *Scolytoplatypus* from these collections and provide a DNA-based phylogenetic analysis of several Chinese and other *Scolytoplatypus* species. We also record eight species from mainland China for the first time and provide a key to the males of the Chinese species.

## ﻿Materials and methods

### ﻿Abbreviations used for collections

**HGT** Private collection of Heiko Gebhardt, Tübingen, Germany;

**JXAU**College of Agricultural Sciences, Jiangxi Agricultural University, Nanchang, China;

**LLY** Private collection of Liu, Lan-Yu, Yilan, Taiwan, China;

**NACRC** National Animal Collection Resource Center, Beijing, China;

**RAB** Private collection of Roger A. Beaver, Chiang Mai, Thailand;

**SYU** Museum of Biology, Sun Yat-sen University, Guangzhou, China;

**USNM**National Museum of Natural History, Washington D.C., USA.

Collections were made in Jiangxi, Yunnan, Sichuan, Zhejiang, Fujian, and Chongqing in China. The samples were immediately preserved in tubes containing 99.9% ethyl alcohol, which were stored at – 20 °C for DNA extraction and examination. We used a stereoscopic microscope (Cnoptec SZ680) to examine the beetles. Photographs were taken with Keens Ultra-Depth of Field 3D Microscope (VHX-600). All photographs were further adjusted and assembled with Adobe Photoshop CS6. All measurements were made on ten specimens of both sexes chosen to show the entire variability range. The body length was measured from the anterior pronotal margin to the elytral apex. Finally, we also provide references to published illustrations of several species, which can be found on the Internet or in publications.

DNA was extracted from the adult head. The total genomic DNA was extracted from each individual using the Ezup Column Animal Genomic DNA Purification Kit (Sangon Biotech Co. Ltd). Amplification of four gene fragments (COI, EF-1α, CAD, 28S) was made by PCR, using primers and cycling conditions previously described (Jordal et al. 2011). The PCR products containing target bands were sent to Sangon Biotech Co. Ltd (Shanghai, China) for purification and sequencing, and the sequences were analysed using the software DNAstar. Additional information on the *Scolytoplatypus* material was collected by the authors in China or downloaded from **NCBI** (The National Center for Biotechnology Information) (Table 1). Concatenated DNA sequence data from Jordal (2013) were analysed in MrBayes v. 3.2.6, Partitions and models were estimated by PartitionFinder 2 and ModelFinder respectively in PhyloSuite, GTR+G were selected for each partition. 10 million generations were run, with 25% of the generations as burn-in, PSRF close to 1.0 and standard deviation of split frequencies below 0.01 were accepted (Lai et al. 2021). Maximum likelihood (ML) analyses were conducted with IQ-TREE. The optimal model of molecular evolution found by ModelFinder each partition based on the Bayesian information criterion (BIC) scores. Each partition was as follows: 28S = GTR+I+G, CAD = TVMEF+I+G, COI= GTR+I+G, EF-1α = TRN+I+G. Clade support was assessed by 5000 bootstrap pseudoreplicates of the combined data set.

**Table 1.** Material used for phylogenetic analyses, including their GenBank accession numbers.

No.	Taxon	Country	COI	CAD	EF-1ɑ	28S	Reference
1	*Remansusmutabilis*	Madagascar	KF758328	KF758316	KF758341	KF758300	Jordal 2013
2	*R.pygmaeus*	Madagascar	–	KF758310	KF758338	KF758294	Jordal 2013
3	*R.sahondrae*	Madagascar	KF758331	KF758319	KF758347	KF758303	Jordal 2013
4	*Scolytoplatypusafricanus*	Uganda	EU191866	HQ883822	EU191898	AF308391	Jordal 2013
5	*brahma*	China: Yunnan	LC657958	LC657966	LC657974	LC657950	This study
6	*S.calvus*	China: Yunnan	LC657960	LC657968	LC657976	LC657952	This study
7	*S.congonus*	Tanzania	KF758322	KF758306	KF758334	KF758290	Jordal 2013
8	*S.entomoides*	Papua New Guinea	HQ883679	HQ883823	HQ883748	–	Jordal 2013
9	*S.fasciatus*	South Africa	KF758324	KF758309	KF758337	KF758293	Jordal 2013
10	*S.hova*	Madagascar	KF758326	KF758314	KF758340	KF758298	Jordal 2013
11	*S.javanus*	Malaysia	KF758333	–	KF758349	KF758305	Jordal 2013
12	*S.minimus*	China: Sichuan	LC657959	LC657967	LC657975	LC657951	This study
13	*S.neglectus*	Cameroon	KF758332	KF758320	KF758348	KF758304	Jordal 2013
14	*S.permirus*	Madagascar	KF758325	KF758311	KF758339	KF758295	Jordal 2013
15	*S.pubescens*	China: Yunnan	LC657956	LC657964	LC657972	LC657948	This study
16	*S.raja*	China: Yunnan	LC657954	LC657962	LC657970	LC657946	This study
17	*S.rugosus*	Madagascar	KF758330	KF758317ext-link>	KF758345	KF758301	Jordal 2013
18	*S.sinensis*	China: Jiangxi	LC657957	LC657965	LC657973	LC657949	This study
19	*S.truncatus*	Cameroon	KF758323	KF758308	KF758336	KF758292	Jordal 2013
20	*S.tycon*	Japan	JF894375	–	JF713688	JX263764	Jordal 2013
21	*S.unipilus*	Gabon	MG979488	MG979490	MG979489	–	Jordal 2013
22	*S.wugongshanensis* sp. nov.	China: Jiangxi	LC657953	LC657961	LC657969	LC657945	This study
23	*S.skyliuae* sp. nov.	China: Jiangxi	LC657955	LC657963	LC657971	LC657947	This study

## ﻿Taxonomic account

### ﻿ Scolytoplatypus wugongshanensis

Taxon classificationAnimaliaColeopteraCurculionidae

Liao, Lai & Beaver sp. nov.

39B13B0A-C8B1-5836-84A6-B9DF1C75A614

http://zoobank.org/5D6F7CDD-65BE-4EDF-B594-E61DFC226527

Figure 1

#### Type material.

***Holotype.*** Male, China: Jiangxi Province, Pingxiang City, Luxi County, Wanlongshan Town, Wugong Mountain, Yangshimu, 27°34'47"N, 114°13'57"E, 27.IX.2017, log dissection, host Fagaceae sp, Shengchang Lai leg. (deposited in NACRC).

***Allotype.*** Female, the same data as the holotype (deposited in NACRC).

***Paratypes.*** 10 males, 10 females, the same data as the holotype (8 males, 8 females JXAU; 2 males, 2 females NACRC); 3 males, China: Jiangxi Province, Ji’an City, Jinggangshan national nature reserve of Jiangxi, Luofu reservoir, 26°38'27"N, 114°8'45"E, 21.V.2017, log dissection, host unclear, Shengchang Lai leg. (deposited in JXAU); 4 males, 3 females, China: Jiangxi Province, Ganzhou City, Xunwu County, Xiangshan Town, Congkeng Village, 24°55'51"N, 115°51'14"E, 20.V.2018, log dissection, host unclear, Shengchang Lai leg. (1 male, 1 female RAB; 3 males, 2 females JXAU); 17 males, 17 females, China: Fujian Province, Nanping City, Jianyang District, Huilong Town, 27°28'31"N, 118°24'35'E, 152.7m, 15.VIII.2020, log dissection, host unclear, Ling Zhang, Yufeng Cao leg. (2 males, 2 females USNM; 2 males, 2 female LLY; 2 males, 2 females NACRC; 2 males, 2 females RAB; 2 male, 2 females SYU; 7 males, 7 females JXAU).

#### Diagnosis.

**Figure 1.**
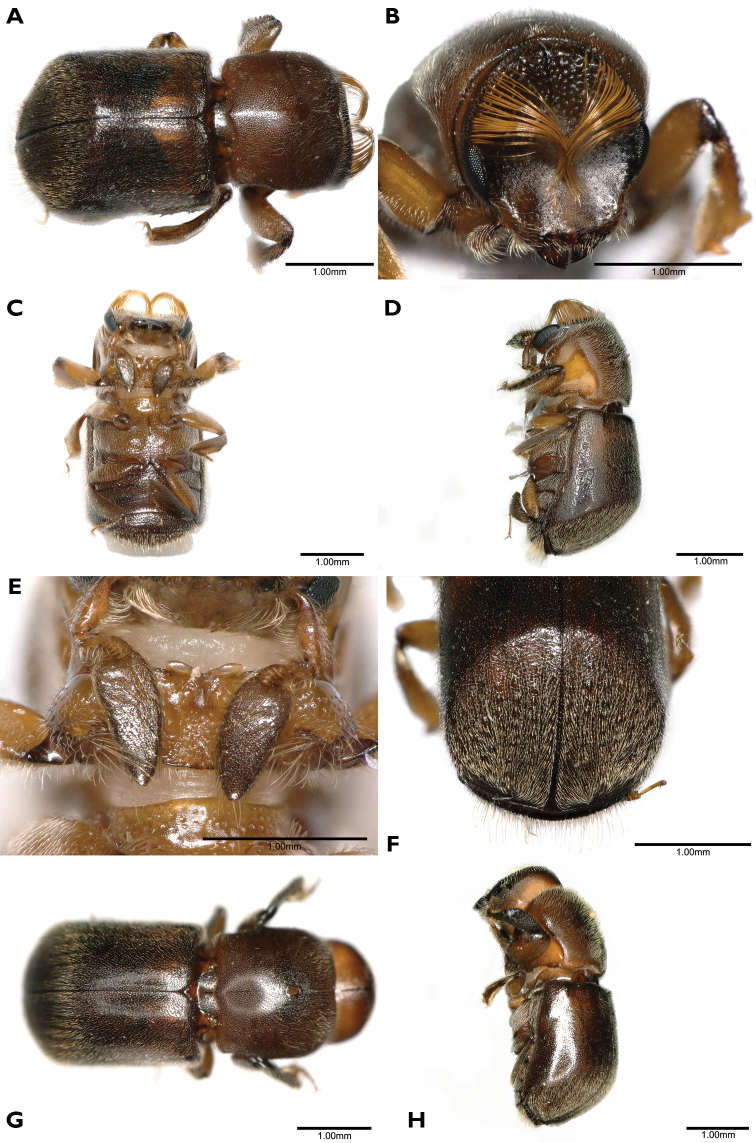
*Scolytoplatypuswugongshanensis* (**A–F** male **G, H** female) **A** dorsal view **B** head anterior view **C** ventral view **D** lateral view **E** prosternum **F** elytral declivity **G** dorsal view **H** lateral view.

The morphology of the species, especially the male prosternum, indicates that it is more closely related to *S.blandfordi* Gebhardt. Both species have the male prosternum raised in a triangle with the apex anterior, and on the anterior margin, two small, flattened processes set close together on each side of the midline (compare Fig. 1E with Beaver and Gebhardt 2006: fig. 2B). Males of *S.wugongshanensis* and *S.blandfordi* can be distinguished using the characters given in Table 2.

**Figure 2.**
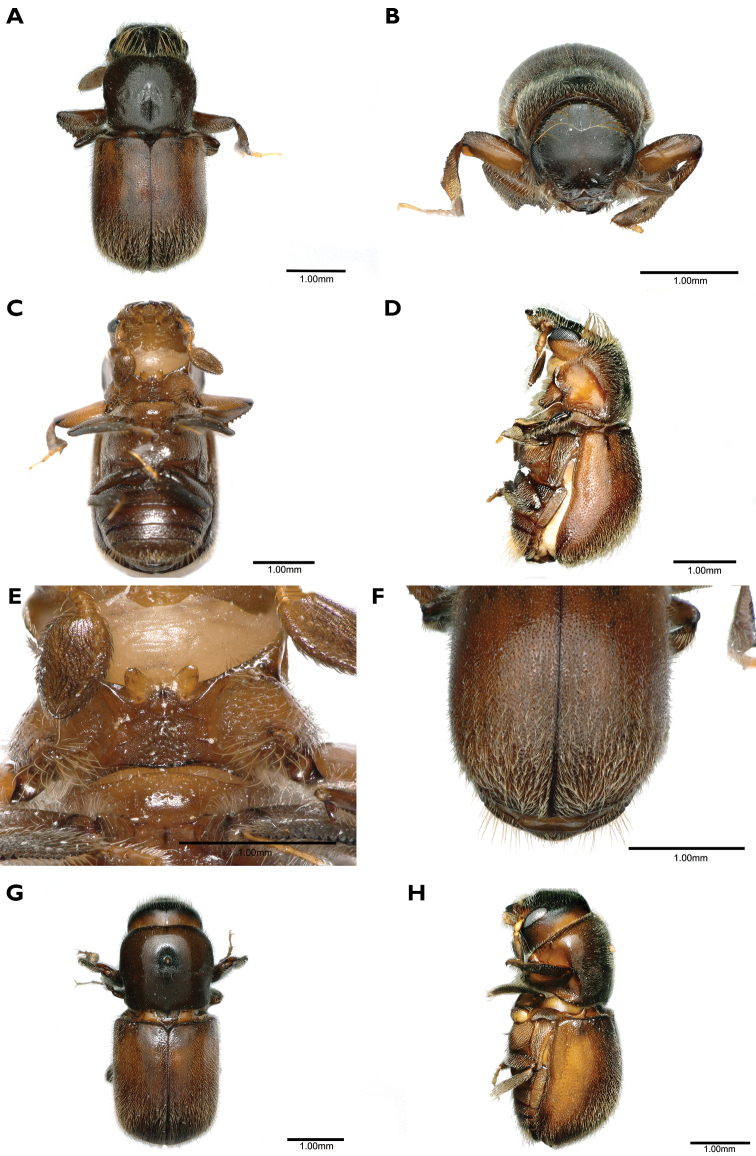
*Scolytoplatypusskyliuae* (**A–F** male **G, H** female) **A** dorsal view **B** head anterior view **C** ventral view **D** lateral view **E** prosternum **F** elytral declivity **G** dorsal view **H** lateral view.

**Table 2.** Diagnostic characters separating *Scolytoplatypuswugongshanensis*, *Scolytoplatypusblandfordi* and *Scolytoplatypusskyliuae*.

	*S.wugongshanensis*	*S.blandfordi*	*S.skyliuae*
Body size	Male 3.8–4.2 mm long; female 4.0–4.6 mm long.	Male 3.1–3.3 mm long; female 3.1–3.3 mm long.	Male 4.0–4.3 mm long; female 4.4–4.8 mm long.
Frons	Male frons with two distinct brushes of long hairs on the margins.	Male frons with a sparse fringe of long hairs.	Male frontal margin with a fringe of hairs dorsally extending to vertex.
Anterior processes of male prosternum	Processes short, bluntly rounded at tip, lying parallel to anterior margin.	Processes longer, falcate, sharply tipped, curving anteriorly.	Processes inserted just behind anterior margin, broadly triangular, diverging at an angle of ~ 90°
Elytral disc	Angularly separated from declivity	Evenly curving into declivity	Evenly curving into declivity
Elytral declivity	Male declivity with small spines present on interstriae 1–7.	Male declivity with spines present only on interstriae 1, 3, 5–7.	Male declivity with granules present on interstriae 2–5, 8.
	Female declivity with long, dense pubescence.	Female declivity with fine, dense, yellowish setae.	Female declivity with long, dense pubescence.

#### Description.

**Male. *Body.*** Length 3.8–4.2 mm (3.8 mm in holotype), 2.00–2.21× as long as wide (2.00 in holotype); dark brown to black in mature specimens, shiny, elytra slightly darker. Whole body covered with fine, yellowish hairlike setae.

***Frons.*** Shallowly concave, slightly flattened above epistoma, with short and shallow depressions on its sides and a fine median impressed line on upper half, lower flattened part without punctures and setae, upper part with minute punctures bearing fine, erect, yellowish setae, margin with two brushes of long golden hairlike setae above the eye, widely separated dorsally, curved towards centre of frons and extending nearly to epistoma; a few long setae on frontal margin at level of antennal insertions.

***Antennal club.*** 2.1–2.2× as long as wide, elongate and triangular, widest near the base, acuminate, densely covered with short appressed setae, anteroventral margin in basal half with a row of seven long, erect setae, thickened and curved at tips, apex with a few long, erect setae.

***Pronotum.*** 0.81–0.87× as long as wide (0.81 in holotype), widest in the middle of its length, anterior margin with distinct median emargination, posterior margin bisinuate, slightly produced in the middle, posterolateral corners approximately rectangular, dorsal surface shining, minutely, moderately densely, rather irregularly punctured except for small, median, impunctate area just anterior to middle of pronotum, corresponding to site of mycangium in female, vestiture of very fine and short hairlike setae. Anteroventral angles with a deep, oval fovea, extending to anterior but not ventral margin of pronotum.

***Prosternum.*** Median part raised in a triangle, its apex anterior not reaching the anterior margin, anterior quarter shining, smooth, the extreme tip sharply pointed, posterior three-quarters of triangle more coarsely granulate. Each granule with a moderately long, backwardly directed seta; anterior margin of prosternum with two symmetrical, divergent, triangular, translucent processes.

***Procoxa.*** Slightly flattened anteriorly, rugose, a group of longer setae near the anterior margin; posteriorly with a small, raised, granulate process bearing a loose brush of long, medially curved, coarse, yellow setae.

***Elytra.*** 1.10–1.21× as long as wide (1.15 in holotype), 1.62–1.76× as long as pronotum (1.69 in holotype), clearly wider than pronotum, sides almost parallel, widest in posterior part, then strongly converging to rounded apex, disc of elytra shining, finely, densely, confusedly punctured, with short, fine, semi-erect, posterior pubescence, declivity angularly separated from disc, striae and interstriae more clearly distinct on declivity, striae broadly but weakly impressed, interstriae very densely, shallowly punctured, interstriae 1–7 each with a row of minute, backwardly directed spines, vestiture longer and denser than elytral disc.

***Abdomen.*** Ventrites shallowly, densely punctured, each puncture with a fine, backwardly directed seta, setae variable in length; last visible ventrite with a band of long golden setae directed posteriorly.

**Female.** (Fig. 1G, H). Length 4.0–4.6 mm (4.0 mm in allotype), 2.11–2.42× as long as wide (2.11 in allotype). Similar to male, slightly larger. Frons triangularly impressed above epistoma, otherwise convex, matt, moderately densely, finely punctured, reticulate between punctures which bear fine, white, erect, hairlike setae, median cranial suture prominent, extending as a fine line to apex of frontal impression. Antennal scape shorter and antennal club oval, shorter and wider than male, rounded at apex, without a row of erect setae antero-ventrally. Pronotum generally as male, but with oval mycangial pit surrounded by erect yellow setae in midline anterior to middle; anteroventral fovea absent. Prosternum a flattened plate lacking specific characters. Procoxae flattened without a process or brush of hairs posteriorly. Elytra generally as in male, but interstrial spines on declivity smaller.

#### Host.

Fagaceae sp.

#### Distribution.

China: Fujian (Nanping) and Jiangxi (Ganzhou, Pingxiang).

#### Biology.

Specimens were collected from small branches (2.0–2.3 mm diameter) of broadleaved trees, including an unidentified species of Fagaceae. The maternal gallery penetrates almost through the whole diameter of the twig, and pupal chambers lie perpendicular to the maternal gallery.

#### Etymology.

The specific name refers to the type locality, Wugongshan Mountain.

### ﻿ Scolytoplatypus skyliuae

Taxon classificationAnimaliaColeopteraCurculionidae

Liao, Lai & Beaver sp. nov.

297C0946-0335-5223-B3F6-9E7838028324

http://zoobank.org/2DCE3543-8434-49A8-8CCC-DD6B0D07BB40

Figure 2

#### Type material.

***Holotype.*** Male, China: Jiangxi Province, Shangrao City, Yanshan County, Wuyishan national nature reserve of Jiangxi, Huanggang Mountain, 27°52'56"N, 117°46'37"E, 17.VII.2017, log dissection, host unclear, Shang Tian, Shengchang Lai, Lifang Xiao & Peishan He leg. (deposited in NACRC).

***Allotype.*** Female, the same data as the holotype (deposited in NACRC).

***Paratypes.*** 8 males, 8 females, the same data as the holotype (6 males, 6 females JXAU; 2 males, 2 females NACRC); 25 males, 19 females, China: Fujian province, Wuyishan city, Wuyishan national nature reserve of Fujian, Guadun Village, 27°44'34"N, 117°38'2"E, 9.VII.2018, 1347.1m, log dissection, host *Castanopsisfargesii* Franch., Shengchang Lai, Kaiping Hu, Jia Lv & Ling Zhang leg. (2 males, 2 females USNM ; 2 males, 2 females LLY; 2 males, 2 females NACRC; 2 males, 2 females RAB; 2 males, 2 females SYU; 15 males, 9 females JXAU).

#### Diagnosis.

Like *S.wugongshanensis*, this species is similar to *S.blandfordi* in its general form and in the structure of the prosternum. The males of those can be distinguished using the characters given in Table 2.

#### Description.

**Male. *Body.*** Length 4.0–4.3 mm (4.0 mm in holotype), 2.11–2.26× as long as wide (2.11 in holotype); dark brown to black in mature specimens, whole body covered with fine, yellowish hairlike setae.

***Frons.*** Strongly concave, slightly flattened above epistoma, with small, shallow depressions at sides, surface minutely reticulate, finely, sparsely punctured, punctures with erect, fine hairs, median line extending ~ 1/4 of frontal height, margins with a row of longer setae below eyes, above eyes a fringe of long, golden setae, extending to vertex, and inwardly curved to middle of frons.

***Antennal club.*** Ovate, ~ 1.7× longer than wide, widest ~ 1/4 length from base, apex narrowly rounded, densely covered with short, appressed setae, anteroventral margin with a row of five or six long erect setae with thickened and incurved tips.

***Pronotum.*** 0.94–1.00× as long as wide (0.94 in holotype), widest at middle, narrowed posteriorly, anterior margin with distinct median emargination, posterior margin bisinuate, slightly produced in the middle, posterolateral corners approximately rectangular, surface smooth, shining, with fine, shallow, irregularly spaced punctures, more densely placed towards posterior margin, bearing fine setae. Anteroventral angles with a deep, oval fovea, not extending to anterior or ventral margins of pronotum.

***Prosternum.*** Median part raised in a triangle, its apex anterior, sharply pointed, not reaching the anterior margin, anterior tip shining, impunctate, posterior part rugose, shallowly punctured, the punctures with appressed, backwardly directed setae. Two symmetrical, triangular, translucent processes diverging at an angle of ~ 90°, inserted just behind anterior margin.

***Procoxae.*** Anterior part flattened, rugose, coarsely, shallowly punctured; posteriorly with a raised, granulate process bearing long, coarse setae, not forming a distinct brush.

***Elytra.*** 1.10–1.16× as long as wide (1.16 in holotype), 1.31–1.47× as long as pronotum (1.37 in holotype), clearly wider than pronotum, sides almost parallel, widest in posterior part, then strongly converging to rounded apex; disc of elytra shining with confused, fine punctures, more closely placed towards declivity, pubescence fine and short, semi-erect, posterior; disc evenly rounded into declivity; declivity convex, densely, finely punctured, sutural interstriae weakly raised in mid-declivity bearing a row of small pointed granules, striae 1 and 2 and interstriae 2 slightly impressed, interstriae 2 without granules except for one or two at top of declivity, interstriae 3 slightly raised with a row of pointed granules, interstriae 4 and 5 with a few scattered granules, interstriae 8 finely carinate posteriorly, the carina extending to the elytral apex, with a row of minute sharply pointed granules posterolaterally; pubescence denser and longer on the declivity then elytral disc .

***Abdomen.*** Ventrites shallowly, densely punctured, each puncture with a fine, backwardly directed seta, setae variable in length; last visible ventrite with a band of long golden setae directed posteriorly.

**Female.** (Fig. 2G, H). Length 4.4–4.8 mm (4.6 mm in allotype), 2.32–2.53× as long as wide (2.42 in allotype). Similar to male, but slightly larger. Frons convex, a weak, triangular impression above epistoma, surface finely reticulate, sparsely, finely punctured, punctures bearing short, fine, erect setae, median cranial suture extending as a median line to apex of triangular impression. Antennal scape shorter than in male, antennal club oval, shorter and wider than male, without a row of erect setae antero-ventrally. Pronotum generally as male, but with a median oval mycangial pit surrounded by erect yellow setae anterior to middle; anteroventral fovea absent. Prosternum a flattened plate lacking specific characters. Procoxae flattened without a posterior process. Elytral declivity generally as in male, but sutural interstriae very slightly raised, and impression lateral to it obsolescent, interstrial granules minute.

#### Host.

*Castanopsisfargesii* Franch. (Fagaceae).

#### Distribution.

Fujian (Wuyishan) and Jiangxi (Shangrao).

#### Etymology.

The species is named for Dr. Sky Liu Lan-Yu for her contributions to the systematics and biology of wood-boring beetles.

#### Molecular data.

**Figure 3.**
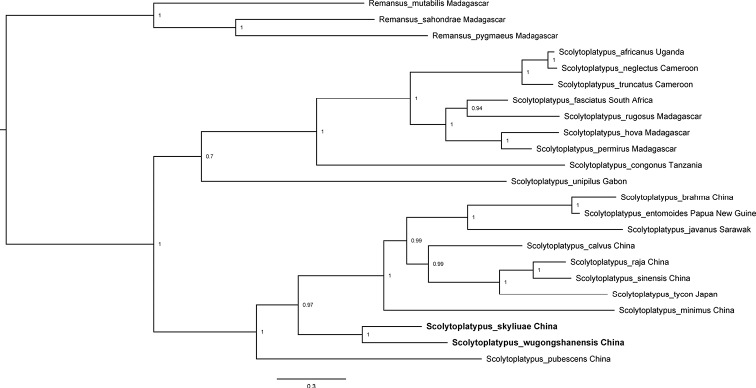
Tree topology resulting from Bayesian and ML analyses of four gene fragments.

The tree topology resulting from the Bayesian and ML analyses of the combined molecular data were near identical and all nodes except one received high support (Fig. 3). Additionally, *S.wugongshanensis* and *S.skyliuae* formed a sister clade to the Asian species of *Scolytoplatypus*. Phylogenetic analysis indicates a rather isolated position for both new species, although their genetic relationship was close.

##### ﻿New records and notes on species

### ﻿ Scolytoplatypus sinensis

Taxon classificationAnimaliaColeopteraCurculionidae

Tsai & Huang, 1965, revised status

01091AC4-0E10-5765-B471-AAB5BF81D36F

Figure 4

Scolytoplatypus sinensis Tsai & Huang, 1965: 121.

#### Taxonomy.

This species has been considered to be a synonym of *S.mikado* Blandford (Wood 1989; Beaver and Gebhardt 2006). Having now examined a larger number of specimens from various provinces of China (including Taiwan), we believe it to be a distinct species. Park (2016) in an unpublished thesis came to the same conclusion based on morphology and DNA differences, but has not officially published his results. Therefore, we resurrect the species here. The species was previously recorded from China (Fujian, Sichuan), Korea and Taiwan (Tsai and Huang 1965; Park 2016). Park (2016) also includes Japan, but we have seen no specimens from that country, nor any published records.

#### Material examined.

China: Jiangxi, Ganzhou, Longnan, Jiulanshan Mtn Nat. Res., 24.622N, 114.564E, 440 m, 17.v.2018, Lai, S-C. (1m, 1f RAB); 5 males 5 females (JXAU) as previous except: Chongyi County, Yangmingshan Forest Park, 25°33'56"N, 114°20'18"E, 10.VII.2021, log dissection, *Cinnamomumcassia* Presl, Ye Xu leg.; China: Yunnan, Honghe, Hani Yi Auton, Pref., Jinping Co., 22.78N, 103.23E, 30.vii.2019, A-Hong-chang, Duan-bo (1m, 1f RAB); China: Zhejiang, Gutianshan Nat. N. Res., 29°8'18”–29°17'29"N, 118°2'14”–118°11'12"E. CSP 21–SE5, 2009, [no collector] (1f RAB); as previous except: 29°25'N, 118°12'E, 402 m, CSP 13–SE4, 2010 (1m RAB); 7 males, 7 females (JXAU) China: Fujian Province, Zhangzhou City, Yunxiao County, Wushan, 23°55'20"N, 117°11'31"E, ca 740 m, 15.VII.2019, log dissection, *Cinnamomumcamphora* (L.) Presl, Song Liao, Ling Zhang & Shengchang Lai leg.; 14 males, 20 females (JXAU) China: Chongqing, Wushan County, Luoping Town, 31°12'23"N, 110°05'18"E, 21.VIII.2016, log dissection, host unclear, Shang Tian leg.; 43 males, 27 females (JXAU) China: Sichuan Province, Xichang City, Leibo County, Mahu Township, 28°24'54"N, 103°46'16"E, ca 1088 m, 5.VIII.2021, log dissection, *C.camphora*, Song Liao leg.

#### Distribution.

China (Chongqing, Fujian, Jiangxi, Sichuan, Yunnan, Zhejiang)

#### Remarks.

The characters given by Tsai and Huang (1965) to distinguish the species from *S.mikado* are not entirely reliable, showing some degree of intraspecific variation, and their figures can be misleading. The male of *S.sinensis* can be distinguished from *S.mikado* by the following characters (*S.sinensis* given first): Prosternum strongly humped anteriorly when viewed from below vs. anterior part of prosternum almost flat or weakly raised, never strongly humped; anterior processes of prosternum directed forwards, diverging by up to 60° vs. anterior processes directed more laterally, diverging at an angle of 60–120°; procoxae with a dense brush of 15–20 long, erect setae anteriorly near the inner margin vs. procoxae with only a few (4–7) long, erect setae anteriorly; smaller size, male 3.0–3.2 mm long vs. male 3.3–3.6 mm long. It has not been possible to find characters which will reliably separate the females of the two species if they are not collected in association with males. The suggestion of Tsai and Huang (1965) that there is a difference in the position of the mycangial pore on the dorsal surface of the pronotum is not borne out by the examination of numerous specimens of both species.

**Figure 4.**
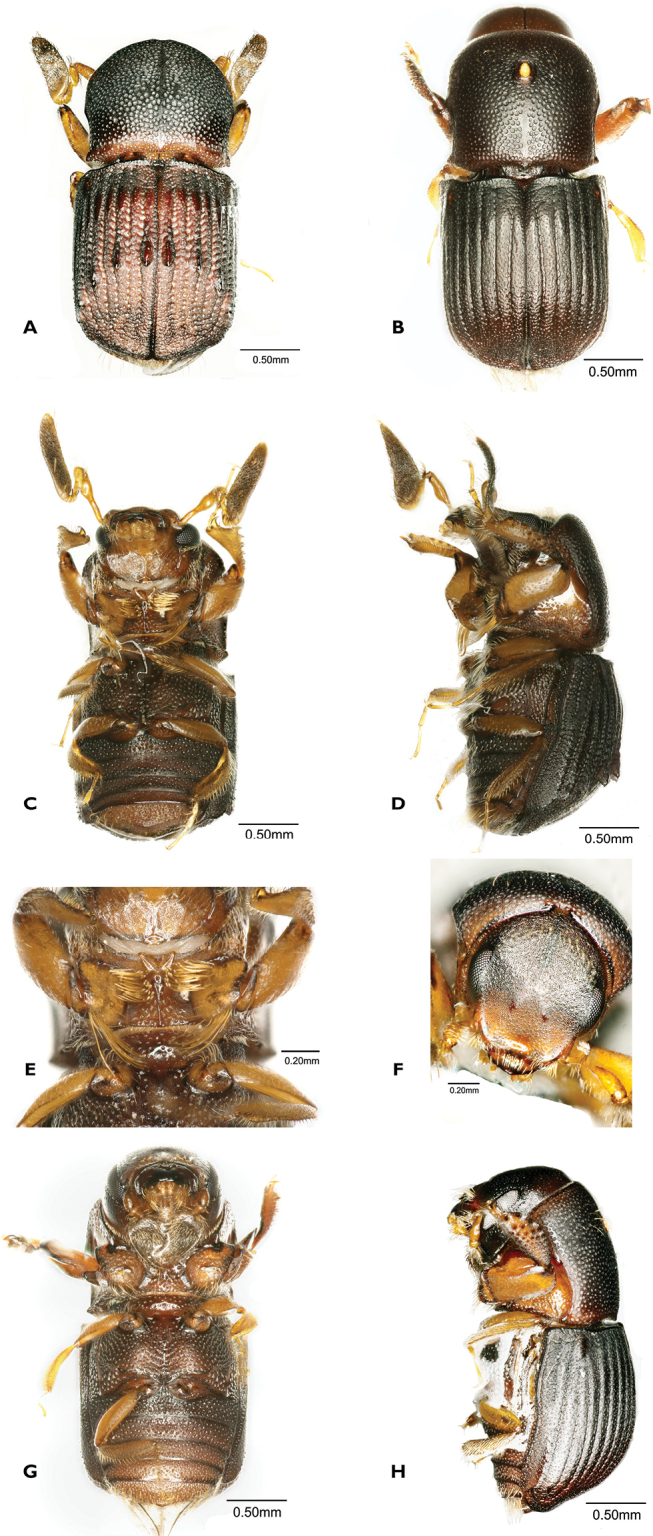
*Scolytoplatypussinensis* (**A, C–F** male **B, G, H** female) **A** dorsal view **B** dorsal view **C** ventral view **D** lateral view **E** prosternum **F** head anterior view **G** ventral view **H** lateral view.

#### Hosts.

*Alnuscremastogyne* Burk. (Betulaceae), *Amygdalusdavidiana* (Carr.) C. de Vos (Rosaceae), *Phoebezhennan* S.K. Lee & F.N.Wei (Lauraceae), Theaceae (Tsai and Huang 1965). *Cinnamomumcamphora* and *C.cassia* (Lauraceae) are newly recorded hosts.

### ﻿ Scolytoplatypus blandfordi

Taxon classificationAnimaliaColeopteraCurculionidae

Gebhardt

F27369C1-D567-5B9A-A04E-455DDCE2EDB9

Scolytoplatypus blandfordi Gebhardt, 2006: 162, fig. 2B.

#### Material examined.

China: Yunnan, Honghe, Maguanm Gulingqin, 22.731N, 103.993E, 592 m, FIT, 24.iv.2018, DJS17, L.Z. Meng (1m RAB); as previous except: Lijiang, 24.143N, 100.227E, 3221 m, 28.v.2018, LJ3200–4FI (2m RAB); as previous except: Puer, Jingdong, Ailoshan, 24.532N, 101.015E, 2499 m, 8.v.2018, ALS(S)2400–3FI (2m RAB); as previous except: 24.517N, 101.012E, 9.vi.2018, FIT, ALS2200–2FI (1f RAB).

#### Distribution.

China (Taiwan). New to Chinese mainland (Yunnan).

#### Diagnosis.

The species is related to *S.wugongshanensis* and *S.skyliuae.* The males can be distinguished by the characters given in Table 2 and the key.

#### Host.

Recorded from *Cyclobalanopsismorii* (Hayata) Schottky (Fagaceae) (Beaver and Gebhardt 2006).

### ﻿ Scolytoplatypus brahma

Taxon classificationAnimaliaColeopteraCurculionidae

Blandford

94BD700B-E839-5600-842B-50BE2B76F172

Figure 5

Scolytoplatypus brahma Blandford, 1898: 425.
Scolytoplatypus hamatus Hagedorn, 1904: 260. Synonymy: Schedl 1952: 159.
Scolytoplatypus hirsutus Blackman, 1943: 124. Synonymy: Schedl 1952: 159.
Scolytoplatypus paucegranulatus Eggers, 1935: 242. Synonymy: Beaver and Gebhardt 2006: 167.

#### Material examined.

4 males, 1 female (JXAU) China: Yunnan Province, Honghe Hani and Yi Autonomous Prefecture, Jinping County, 22°46'48"N, 103°13'48"E, 21.IV.2017, ethanol trap, Bo Duan, Hongchang A leg.; 1 female (JXAU) as previous except: Hekou County, Mahuangbao, 22°34'49"N, 103°58'30"E, 10.VIII.2018, ethanol trap, Bo Duan, Hongchang A leg.; 1 male (JXAU) as previous except: Xishuangbanna Dai Autonomous Prefecture, Mengpeng Farm, 21°24'45"N, 101°20'21"E, 26.VII2018, ethanol trap, Bo Duan, Hongchang A leg.; 1 male (JXAU) as previous except: Mengman Farm, 21°23'34"N, 101°18'51"E, 26.VII.2018, ethanol trap, Bo Duan, Hongchang A leg.; 1 male (JXAU) as previous except: Mengpeng Farm, 21°24'45"N, 101°20'21"E, 17.XI2019, ethanol trap, Bo Duan, Hongchang A leg.

#### Distribution.

Bangladesh, Borneo, India, Indonesia (Sumatra, Java) Malaysia, Thailand (Sabah) (Beaver and Gebhardt 2006; Beaver et al. 2014). New to China (Yunnan)

#### Diagnosis.

The species is most closely related to *S.bombycinus*, but is considerably smaller. The male of *S.brahma* is characterised by its characteristic prosternal plate, which is structurally similar to that of *S.bombycinus*, and by a small elongate swelling in the midline on the upper part of the frons (Blackman 1943: fig. 12).

**Figure 5.**
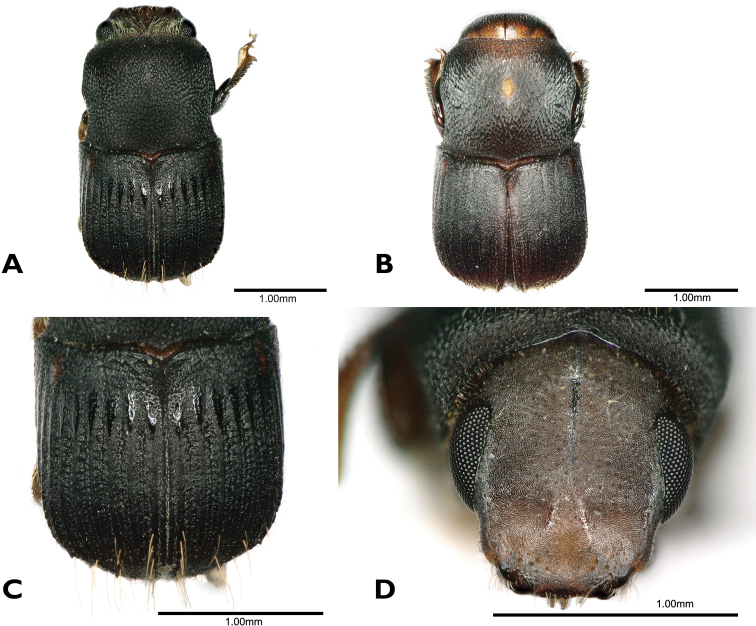
*Scolytoplatypusbrahma* (**A, C, D** male **B** female) **A** dorsal view **B** dorsal view **C** elytral declivity **D** head anterior view.

#### Host.

This species is polyphagous attacking a wide range of host trees in many families, including: *Cryptocaryawightiana* Thwaites (Lauraceae), *Ilexdipyrena* Wall. (Aquifoliaceae), *Swieteniamahagoni* (L.) Jacq. (Meliaceae) (Wood 1992), *Erythrinasubumbrans* (Hassk.) Merr. (Leguminosae), *Heveabrasiliensis* (Willd. ex A. Juss.) Muell. Arg. (Euphorbiaceae), *Theobromacacao* L. (Sterculiaceae), *Vernoniaarborea* Buch.-Ham. (Compositae) (Kalshoven 1959).

### ﻿ Scolytoplatypus calvus

Taxon classificationAnimaliaColeopteraCurculionidae

Beaver & Liu

B8D4AA8E-8280-52DD-B437-CAE7BEBC3456

Figure 6

Scolytoplatypus calvus Beaver & Liu, 2007: 227.

#### Material examined.

China: Sichuan Prov., Moxi env., Hailuogou valleu, Gonghe vill., 29°37'27"N, 102°06'28"E, 1715 m, 17–21.vi.2014, at light, J. Hájek, J. Růžička, M. Thoč (1m RAB); China: W Fujian, Emei Feng, 27°01'N, 117°04'E, 1200–1500 m, 3.-4.vi.2008, Jaroslav Turna (1 female HGT); 2 females (JXAU) China: Yunnan Province, Chuxiong City, Zixi Mountain, 25°0'15"N, 101°24'39"E, ca. 2354 m, 12.VIII. 2021, ethanol trap, Song Liao, Guangyu Yu leg.

#### Distribution.

China (Taiwan). New to Chinese mainland (Fujian, Sichuan, Yunnan).

#### Diagnosis.

*S.calvus* is most closely related to *S.mikado* and *S.raja*, but is smaller than either, and may be distinguished by the characters given in the key.

**Figure 6.**
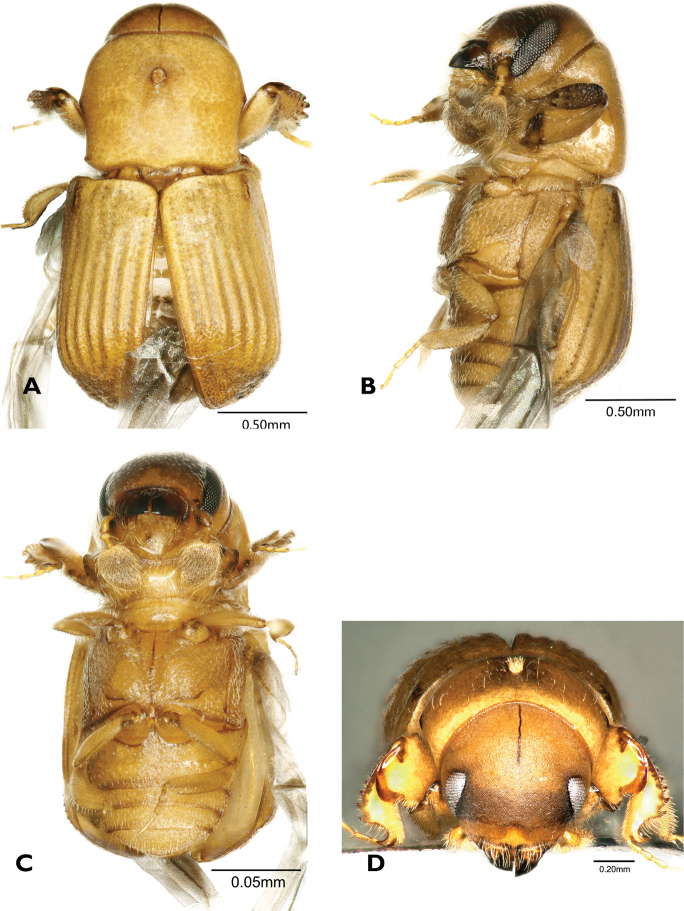
*Scolytoplatypuscalvus* (**A–D** female) **A** dorsal view **B** lateral view **C** ventral view **D** head anterior view.

#### Host.

Not known.

### ﻿ Scolytoplatypus curviciliosus

Taxon classificationAnimaliaColeopteraCurculionidae

Gebhardt

82FFADAC-6D3D-5F3B-80C7-501E9C2C1693

Scolytoplatypus curviciliosus Gebhardt, 2006: 165, fig. 2K.

#### Material examined.

China: Yunnan, Xishuangbanna, 28 km NW Jinghong, vic. An Ma Xi Zhan (NNNR), 22°12'E, 100°38'E, 700 m, forest, EKL, 8.vii.2008, A. Weigel (1m, 6f RAB); as previous except: Mengla, Bubeng, 21.610N, 101.582E, 709 m, 6.iii.2019, BB(S)600–4FI, L.Z. Meng (4m, 4f RAB); as previous except: Menglun, 21.929N, 101.254E, 600 m, 2.iv.2018, XTBG600–1FI (2m, 1f RAB).

#### Distribution.

Philippines, Thailand. New to China (Yunnan).

#### Diagnosis.

The species most closely resembles *S.parvus* and *S.reticulatus*. The male can be distinguished from *S.parvus* by the absence of granules and conspicuous white hairs on the lower part of the elytral declivity, and from *S.reticulatus* by the lack of teeth on the interstriae at the summit of the elytral declivity, and the impressed elytral striae. The females of all three species lack a mycangial pore on the pronotum. The female of *S.curviciliosus* can most easily be distinguished from *S.parvus* by its slightly larger size (2.0–2.1 mm vs. 1.8–1.9 mm in *S.parvus*), and the more strongly angulate posterior angles of the pronotum, and from *S.reticulatus* by the non-impressed elytral striae, obsolescent on the declivity, and flat, not convex, interstriae (Beaver and Gebhardt 2006).

#### Host.

Unclear.

### ﻿ Scolytoplatypus minimus

Taxon classificationAnimaliaColeopteraCurculionidae

Hagedorn

D47496B8-37FE-5F00-9D48-2D5C0337DC1A

Figure 7

Scolytoplatypus minimus Hagedorn, 1904: 125.

#### Material examined.

1 female (JXAU) China: Yunnan Province, Baoshan City, Gaoligong Mountain Nature Reserve, Baihualing, 25°18'22"N, 98°47'45"E, ca. 1600 m, 28.VII.2019, ethanol trap, Song Liao, Shengchang Lai leg.; CHINA: Yunnan, Yulongshan mts., Ganhaizi pass, 3500 m, 18–23.vii.1990, V. Kubáň (1m RAB); as previous except: Xishuangbanna, 28 km NW Jinghong, vic. An Ma Xi Zhan (NNNR), 22°12'E, 100°38'E, 700 m, EKL, 16.iii.2009, L. Meng (1f RAB); as previous except: Mengla, Bubeng, 21.610N, 101.582E, 717 m, 16.iii.2019, BB(X)600–3FI, L.Z. Meng (2f RAB); 17 males, 3 females (JXAU) China: Sichuan Province, Xichang City, Leibo County, Mahu Township, 28°24'54"N, 103°46'16"E, ca. 1088 m, 5.VIII.2021, log dissection, host *Cinnamomumcamphora*, Song Liao leg.; CHINA: Sichuan, Mt. Emei, 600–1050 m, 5–19.v.1989, L. Bocák (4f RAB); as previous except 1000 m, 4–20.v.1979, V. Kubáň (4f RAB); as previous except: [no altitude], viii.2016, Tian-Shang (1m, 1f RAB).

**Figure 7.**
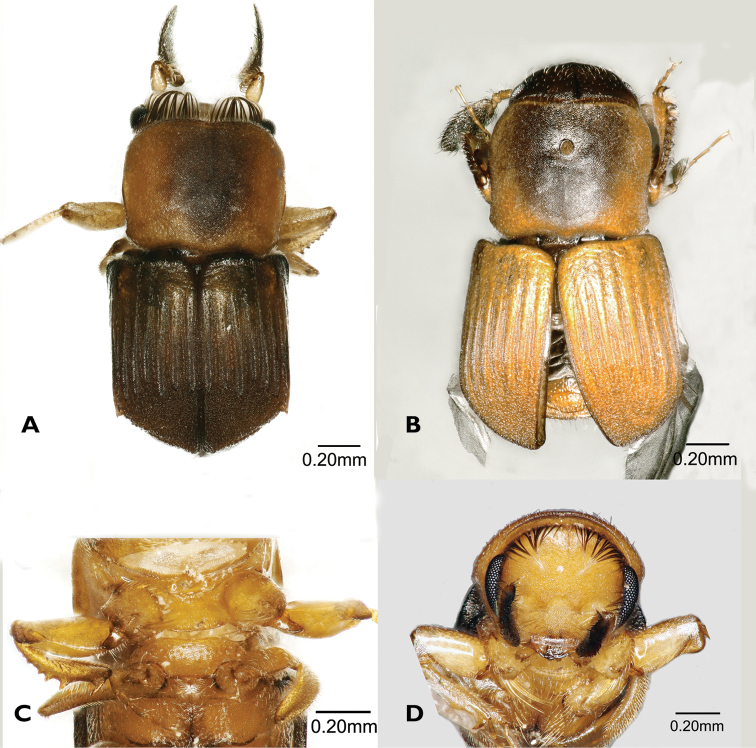
*Scolytoplatypusminimus* (**A, C, D** male **B** female) **A** dorsal view **B** dorsal view **C** prosternum **D** head anterior view.

#### Distribution.

India (Uttar Pradesh, West Bengal); Thailand (Chiang Mai, Nakhon Sri Thammarat) (Beaver and Gebhardt 2006). New to China (Sichuan, Yunnan).

#### Diagnosis.

The male appears to be related to *S.reticulatus* which has a similar prosternum (compare figures 1K and 1L in Beaver and Gebhardt 2006). The females are also very similar, but the female of *S.reticulatus* lacks the typical mycangial pore on the pronotum.

#### Host.

Recorded from species of *Alnus* (Betulaceae), *Cornus* (Cornaceae), and *Prunus* (Rosaceae) (Beeson 1961), *Cinnamomum* (Lauraceae) (Beaver and Browne 1975), and *Acrocarpus* (Leguminosae) (Saha and Maiti 1996). Evidently polyphagous. In Thailand, the species was found in smaller branches (1–2 cm diameter) of *Cinnamomuminers* Reinw. ex Bl. than those attacked by *S.raja* and *S.pubescens* (Beaver and Browne 1975).

### ﻿ Scolytoplatypus pubescens

Taxon classificationAnimaliaColeopteraCurculionidae

Hagedorn

B4D7A107-5821-53CF-8C96-B262D58F8E13

Figure 8

Scolytoplatypus pubescens Hagedorn, 1904: 123.
Scolytoplatypus pubescens kabakovi Axentjev, 1992: 192.

#### Material examined.

China: Sichuan, Mt. Emei, 600–1050 m, 5–19.v.1989, L. Bocák (4m, 2f RAB); as previous except 1000 m, 4–20.v.1979, V. Kubáň (4m, 3f RAB); Yunnan, Baoshan, Gaoligong Nat. Res., Bai-Hua-Ling, 25.306N, 98.796E, ca.1600 m, 8.vii.2019, Lai, S-C. & Liao, S. (1m, 1f RAB); 28 males, 19 females (JXAU) as previous except: 25°17'59"N, 98°47'12"E, ca. 1970 m, 20.VIII.2021, host *Caryacathayensis*, Song Liao, Guangyu Yu leg.; Honghe, Hekou, Dajianshan, 22.909N, 103.697E, 2130 m, FIT, 12.v.2018, DJS4–3S, LZ Meng (1m,1f RAB); Wenshan, Maguan, Gulingqin, 22.732N, 103.994E, 594 m, FIT, 1.iv.2018, GLQ31, LZ Meng (1m RAB); Xishuangbanna, 23 km NW Jinghong, vic. Na Ban village (NNNR), 22°10'N, 100°39'E, 7–1000 m, L. Meng (3f RAB).

#### Distribution.

India (Assam, Uttar Pradesh, West Bengal), Myanmar, Nepal, Thailand, Vietnam (Beaver et al. 2014), China (Taiwan). New to Chinese mainland (Sichuan, Yunnan)

#### Diagnosis.

*S.pubescens* is most closely similar to *S.superciliosus* and *S.zahradniki*, but can be distinguished by the characters given in the key.

#### Host.

The species is known to attack at least 12 different families of trees (Beaver and Gebhardt 2006). *Caryacathayensis* Sarg. (Juglandaceae) is recorded here as a new host.

**Figure 8.**
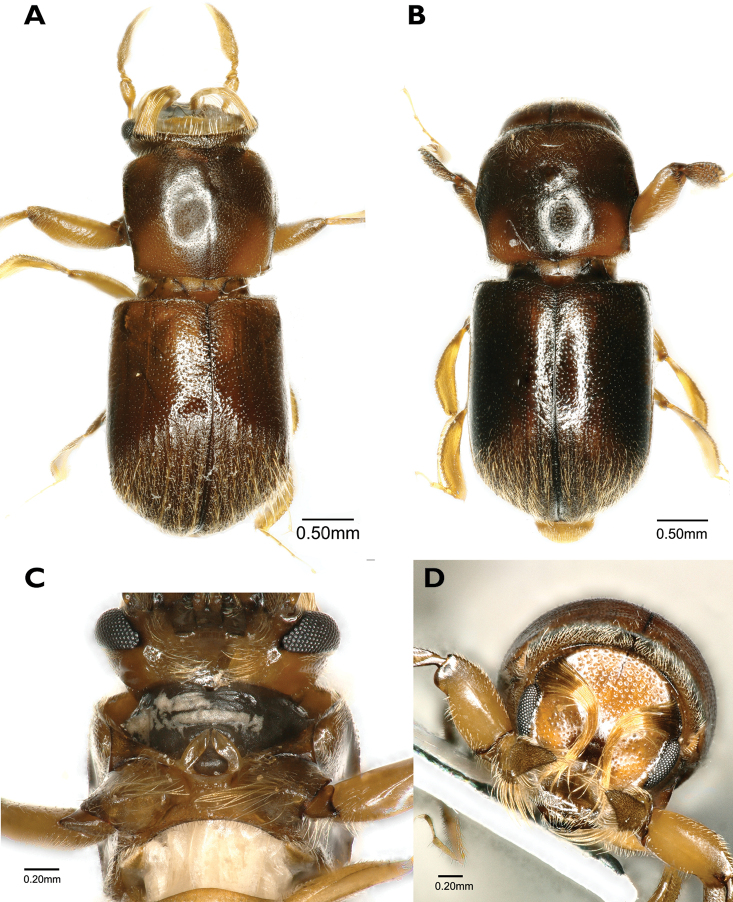
*Scolytoplatypuspubescens* (**A, C, D** male **B** female) **A** dorsal view **B** dorsal view **C** prosternum **D** head anterior view.

### ﻿ Scolytoplatypus ruficauda

Taxon classificationAnimaliaColeopteraCurculionidae

Eggers

81F8D213-FA30-5B58-80D4-0BE4B58793BB

Scolytoplatypus ruficauda Eggers, 1939: 9: Beaver and Gebhardt 2006: fig.2A.

#### Material examined.

CHINA: Yunnan, Puer, Jingdong, Ailoshan, 24.5411N, 101.030E, 2690 m, 8.v.2018, ALS(S)2600–5FI, L.Z. Meng (6m HGT).

#### Distribution.

Myanmar, Nepal (Beaver and Gebhardt 2006). New to China (Yunnan).

#### Diagnosis.

The species is most closely similar to *S.wugongshanensis*. but can be distinguished by the characters given in the key.

#### Host.

Not known.

### ﻿ Scolytoplatypus samsinghensis

Taxon classificationAnimaliaColeopteraCurculionidae

Maiti & Saha

043207EC-14E5-5E40-9733-6A22D498D550

Scolytoplatypus samsinghensis Maiti & Saha, 2009: 101, fig. 20.

#### Material examined.

China: Yunnan, Xishuangbanna, Mengle, Bubeng, 21.615N, 101.579E, 853 m, FIT, 5.v.2018, BB800–1FI, L.Z. Meng (3m RAB); as previous except: 21,611N, 101.581E, 712 m, 15.v.2018. BB600–2FI (3f RAB).

#### Distribution.

India (West Bengal). New to China (Yunnan).

#### Diagnosis.

The species is very close to *S.eutomoides* Blandford, but differs from it on the basis of the following characters of the males (*S.samsinghensis* given first): 1. striae 1 and 2 marked up to the lower half of declivity, little elevation at interstriae 1 and 3, on or near the elevation with fairly large granules vs. striae 1 and 2 marked up to upper half of declivity and lower half with smaller granules; 2. punctures on pronotum rather deep vs. punctures on pronotum, rather shallow; 3. strial groove marked by elongate and confluent punctures vs. strial groove devoid of any distinct punctures; 4. frontal hairs restricted only towards vertex vs. upper half of frons with hairs (Maiti and Saha 2009).

#### Host.

Not known.

### ﻿Key to males of Chinese *Scolytoplatypus* species

**Table:** 

1	Front femur with a tooth above near apex	**2**
–	Front femur not toothed above	**3**
2	Prosternum with median carina, extending from near the base and clearly separating the anterior, concave processes, its tip minutely granulate and setose, extending just beyond the anterior margin of the prosternum. Striae and interstrial carinae becoming obsolescent on declivity. Larger species, 3.7–3.8 mm long	***S.samsinghensis* Maiti & Saha**
–	Prosternum almost flat, the concave, anterior processes almost contiguous in midline, very narrowly separated by a carinate process extending to anterior margin of pronotum, its tip smooth and glabrous. Impressed striae visible almost to apex of elytra. Smaller species, 2.6–3.1 mm long	***S.brahma* Blandford**
3	Small species, not more than 2 mm long	**4**
–	Larger species, more than 2 mm long	**5**
4	Declivity beginning posterior to the middle of elytra. Elytra with distinct teeth on interstriae at summit of declivity, alternately longer and shorter. Frons with an even fringe of hairs around the upper half of the frontal impression. Prosternum with a small triangular median projection anteriorly, behind this a pair of widely separated, weakly shining, flattened areas. 1.4–1.6 mm long	***S.minimus* Hagedorn**
–	Declivity beginning anterior to the middle of elytra. Summit of elytral declivity without denticles. Frons with brushes of long, incurving hairlike setae both above and below eyes. Prosternum flat, smooth, with a small, flat, broadly rounded process anterior. 1.8–1.9 mm long	***S.curviciliosus* Gebhardt**
5	Summit of elytral declivity marked by strongly developed spines on alternate interstriae, the spines projecting over a steep declivity. Basal angles of pronotum strongly produced, pointed apically	**6**
–	Summit of elytral declivity without spines or with small spines on all interstriae. Spines not projecting over a steep declivity, declivity in side view angular or gradually rounded. Basal angles of pronotum not produced	**9**
6	Body length 2.4–2.5 mm. Frons without a dorsal fringe of longer setae. Pronotum minutely punctured. Prosternum with a transverse ridge anteriorly, sometimes bearing two tubercles, and anterior to this a pair of widely separated tapering processes	***S.calvus* Beaver & Liu**
–	Body length 3.0–3.6 mm. Frons with a dorsal fringe of setae. Pronotum coarsely, shallowly punctured. Prosternum with two tubercles anteriorly, and anterior to them a pair of narrowly separated tapering processes	**7**
7	Elytral spines with a few short setae only, elytral declivity glabrous or almost so. 3.0–3.6 mm long	**8**
–	Elytral spines with tufts of long setae, elytral declivity covered with short interstrial hairs. 3.0–3.2 mm long	***S.raja* Blandford**
8	Prosternum strongly humped anteriorly. Anterior prosternal processes directed forwards, diverging by up to 60°. Procoxae with a dense brush of 15–20 long, erect setae anteriorly near the inner margin. 3.0–3.2 mm long	***S.sinensis* Tsai & Huang**
–	Prosternum not strongly humped anteriorly, flat or weakly raised. Anterior prosternal processes directed more laterally, diverging at an angle of 60–120°. Procoxae with only a few (4–7) long, erect setae anteriorly. 3.3–3.6 mm long	***S.mikado* Blandford**
9	Interstriae on posterior part of disc distinctly costate or carinate. Elytra with small interstrial teeth near summit of declivity	**10**
–	Interstriae on posterior part of disc not raised. Elytra without interstrial teeth at summit of declivity (except *pubescens* with minute teeth)	**11**
10	Frons with brushes of hairlike setae above and below eyes. Anterior margin of prosternum projecting in two rounded lobes, slightly asymmetrical, and with a translucent process on the right side only. Pronotum without a deep fovea at the antero-ventral angle. 2.8–3.0 mm long	***S.superciliosus* Tsai & Huang**
–	Frons with a fringe of setae around upper part of frontal impression. Anterior margin of prosternum with two symmetrical, divergent, triangular processes. Pronotum with a deep fovea at the antero-ventral angle. 2.6–3.3 mm long	***S.zahradniki* Knížek**
11	Middle of frons with an area with very dense, short setae. Elytra with minute interstrial teeth at summit of declivity. Prosternum anteriorly with two triangular processes inserted on anterior margin, widely separated at the base but converging towards the midline. 3.5–3.7 mm long	***S.pubescens* Hagedorn**
–	Middle of frons without an area with very dense, short setae. Elytra without teeth at summit of declivity. Prosternum not as above	**12**
12	Prosternum raised in middle in a triangle, the apex anterior or posterior	**13**
–	Prosternum flat or weakly convex, not raised in a triangle	**16**
13	Apex of prosternal triangle anterior, with a single pointed tubercle; anterior margin with two symmetrical, divergent, triangular, translucent processes	**14**
–	Apex of prosternal triangle posterior, anterior margin projecting in two rounded lobes, slightly asymmetrical, and with a translucent process on the right side only. 2.8–3.0 mm long	***S.ruficauda* Eggers**
14	Elytral disc angularly separated from declivity. Anterior processes of prosternum inserted on anterior margin, small, short, bluntly rounded at tip, lying parallel to anterior margin. 3.8–4.2 mm long	***S.wugongshanensis* Liao, Lai & Beaver, sp. nov.**
–	Elytral disc evenly curving into declivity. Prosternal processes inserted just behind anterior margin, not as above	**15**
15	Anterior processes of prosternum falcate, sharply tipped and curving anteriorly. Smaller species, 3.1–3.3 mm long	***S.blandfordi* Gebhardt**
–	Anterior processes of prosternum broadly triangular, diverging at an angle of ~ 90°. Larger species, 4.0–4.3 mm long	***S.skyliuae* Liao, Lai & Beaver, sp. nov.**
16	Upper half of frons clearly punctured, lower half impunctate, with a rather sparse fringe of hairs on each side curving inwardly, but not extending to lower half of frons. Prosternum without a pair of translucent processes anteriorly. Rows of punctures on elytral disc feebly but clearly impressed before declivity. Larger species, 3.5–4.5 mm long	***S.tycon* Blandford**
–	Frons impunctate throughout, the incurved brushes of hairs denser and longer, extending beyond middle of frons. Prosternum with a pair of widely separated, translucent, divergent processes anteriorly. Rows of punctures on elytral disc not impressed, usually indistinct. Smaller species, 2.9–3.2 mm long	***S.darjeelingi* Stebbing**

## ﻿Conclusions

Although the biology of the two new species has not been systematically studied, it can be expected to be similar to that of other species of *Scolytoplatypus* (Beaver and Gebhardt 2006). *Scolytoplatypus* is a very characteristic genus of ambrosia beetles, and even the smallest of the known species are larger than the average wood boring beetle (Jordal 2018). The species of *Scolytoplatypus* show marked sexual dimorphism. Some morphological characteristics of Asian species, as opposed to African species, differ between the sexes, e.g., antenna, pronotum, male prosternum. The characters of the male prosternum are particularly useful at specific level in the Oriental species (Beaver and Gebhardt 2006).

The total number of species, host plants and distribution of *Scolytoplatypus* in China are still unclear and need further study. Many other species have been reported in neighbouring countries of China, which still have not been found in China. In addition, new species of *Scolytoplatypus* are being described one after another (Beaver and Gebhardt 2006; Browne 1971; Jordal 2013, 2018; Knížek 2008), indicating quite strongly that many more species remain to be discovered, especially on the mainland China.

## Supplementary Material

XML Treatment for Scolytoplatypus wugongshanensis

XML Treatment for Scolytoplatypus skyliuae

XML Treatment for Scolytoplatypus sinensis

XML Treatment for Scolytoplatypus blandfordi

XML Treatment for Scolytoplatypus brahma

XML Treatment for Scolytoplatypus calvus

XML Treatment for Scolytoplatypus curviciliosus

XML Treatment for Scolytoplatypus minimus

XML Treatment for Scolytoplatypus pubescens

XML Treatment for Scolytoplatypus ruficauda

XML Treatment for Scolytoplatypus samsinghensis
